# A Lactose‐Derived CRISPR/Cas9 Delivery System for Efficient Genome Editing In Vivo to Treat Orthotopic Hepatocellular Carcinoma

**DOI:** 10.1002/advs.202001424

**Published:** 2020-07-21

**Authors:** Yu Qi, Yanli Liu, Bingran Yu, Yang Hu, Nasha Zhang, Yan Zheng, Ming Yang, Fu‐Jian Xu

**Affiliations:** ^1^ State Key Laboratory of Chemical Resource Engineering Key Lab of Biomedical Materials of Natural Macromolecules (Beijing University of Chemical Technology Ministry of Education) Beijing Laboratory of Biomedical Materials Beijing Advanced Innovation Center for Soft Matter Science and Engineering Beijing University of Chemical Technology Beijing 100029 P. R. China; ^2^ Shandong Provincial Key Laboratory of Radiation Oncology Cancer Research Center Shandong Cancer Hospital and Institute Shandong First Medical University Shandong Academy of Medical Sciences Jinan 250117 P. R. China

**Keywords:** biopolymers, CRISPR/Cas9, delivery vectors, lactose, orthotopic hepatocellular carcinoma

## Abstract

Gene editing is a crucial and effective strategy to treat genetic diseases. Safe and effective delivery vectors are specially required for efficient gene editing in vivo of CRISPR/Cas9 system. Interestingly, lactose, a natural saccharide, can specifically bind to asialoglycoprotein receptors, highly expressed on the surface of hepatocellular carcinoma (HCC) cells. Herein, a lactose‐derived branched cationic biopolymer (LBP) with plentiful reducible disulfide linkages and hydroxyl groups is proposed as a potential delivery vector of CRISPR/Cas9 system for efficient genome editing in vivo to treat orthotopic HCC. LBP is synthesized via a facile one‐pot ring‐opening reaction. LBP possesses excellent compacting ability, degradability, biocompatibility, gene transfection performances, and HCC‐targeting ability. LBP‐mediated delivery of classical pCas9‐survivin, which can target and knockout *survivin* oncogene, produces efficient gene editing performances, and superb anti‐cancer activities in orthotopic HCC mouse models. This study provides an attractive and safe strategy for the rational design of CRISPR/Cas9 delivery system.

## Graphical abstract

A polyhydroxy, reduction‐responsive degradable lactose‐derived branched cationic biopolymer is proposed as a safe and effective CRISPR/Cas9 delivery system for targeted therapy in orthotopic hepatocellular carcinoma.

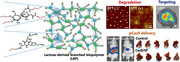

## 1 Introduction

There are more than three thousand human genes with a mutation known to be associated with a genetic disorder or disease phenotype including cancers.^[^ ^1^, ^2^, ^3^, ^4^ ^]^ As a result, new approaches which enable site‐specific editing of human genes show great potential for treating genetic diseases, particularly those which have shown a poor prognosis using conventional treatments, such as chemotherapy or traditional gene therapy.^[^ ^1^ ^]^ The type II bacterial clustered, regularly interspaced, short palindromic repeats (CRISPR)‐associated protein 9 (Cas9) is known as CRISPR/Cas9 system which was originally found in bacteria as an adaptive immune system. Cas9, a nuclease of the *Streptococcus pyogenes* CRISPR/Cas system, can be directed by a short guide RNA (gRNA) to generate site‐specific double‐stranded breaks in almost any human genomic locus of interest.^[^ ^5^, ^6^, ^7^, ^8^ ^]^ In contrast to other traditional non‐nuclease technologies that rely on homologous recombination, the absolute efficiencies of CRISPR/Cas9‐mediated alterations are dramatically higher. Therefore, the CRISPR/Cas9 genome editing system offers unparalleled opportunities for biomedical applications, providing novel therapeutic potentials that are not limited to correcting single‐base mutation of genes causing genetic disorders, but also could be extended to cancer therapy.^[^ ^6^, ^8^, ^9^, ^10^ ^]^ In spite of the high therapeutic relevance of CRISPR/Cas9 system, it is still challenging how to safely and efficiently deliver the genome‐editing Cas9 nuclease to the nucleus of target cells.^[^ ^9^, ^10^ ^]^ Viral vectors are most frequently used for the delivery of *Cas9* nuclease gene. However, the clinical translation potential of viral vectors remains limited, mostly due to the possible long‐term toxicity associated with the risk of integrating virus sequence into human genome.^[^ ^11^ ^]^ Non‐virus delivery of the CRISPR/Cas9 system might be a promising way for its therapeutic application,^[^ ^12^, ^13^, ^14^, ^15^, ^16^, ^17^ ^]^ especially considering the difficulty of packing the *Cas9* gene with large size (about 4.2 kb) into a viral vector. Additionally, the employment of non‐virus vectors would significantly decline the risk of integrating virus sequence into human genome.^[^ ^18^, ^19^, ^20^ ^]^

Natural saccharides‐based cationic gene carriers have been widely studied due to their good biocompatibility and unique biological activity.^[^ ^21^, ^22^, ^23^, ^24^, ^25^ ^]^ Lactose is a natural disaccharide, which contains one glucose residue and one galactose residue. It has been found that asialoglycoprotein receptor (ASGPr), a C‐type lectin primarily expressed on the surface of liver parenchymal cells and hepatic cancer cells,^[^ ^26^ ^]^ could selectively bind to the galactose residue of lactose and facilitate the process of endocytosis.^[^ ^27^, ^28^, ^29^ ^]^ Therefore, taking advantage of ASGPr‐mediated targeting property of lactose might strengthen therapeutic effects of liver diseases. In addition, a tactic of fabricating reduction‐responsive degradable cationic vectors based on the introduction of disulfide linkages has been applied.^[^ ^30^, ^31^, ^32^ ^]^ Disulfide bonds could be broken in the reducing environment to promote the release of nucleic acids and reduce toxicity. It has also been reported that polyhydroxy branched cationic gene carriers have super biocompatible ability and transfection performances.^[^ ^16^, ^31^, ^33^ ^]^ Here we propose a strategy to prepare polyhydroxy, reduction‐responsive degradable lactose‐derived branched cationic biopolymers for delivering CRISPR/Cas9 system to treat orthotopic hepatocellular carcinoma (HCC) via efficient gene editing in vivo.

A lactose‐derived branched cationic biopolymer (LBP) with plentiful disulfide linkages and hydroxyl groups was first synthesized via a facile one‐pot ring‐opening reaction. The biophysical properties, such as compacting ability and reduction‐responsive degradability, were investigated first to determine the possibility of LBP as a potential gene carrier. Subsequently, the cytotoxicity, transfection performances, and ASGPr‐mediated targeting capability of LBP were tested in human BEL7402 HCC cell line. To test the utility of LBP for the delivery of CRISPR/Cas9 genome editing system, in this work we used a well‐known oncogene *survivin* (also called *BIRC5*) as the targeting gene. As a member of the inhibitor of apoptosis protein (IAP) family,^[^ ^34^, ^35^ ^]^ survivin is known as a dual role protein which directly regulates both apoptosis and mitosis in cancer cells during tumorigenesis and tumor metastasis.^[^ ^34^, ^35^ ^]^ *Survivin* is highly expressed in almost all cancers including HCC, but it is undetectable in normal adult tissues. Therefore, *survivin* has been proposed as an attractive target for new anti‐cancer intervention. Interestingly, we observed the obvious liver targeting ability of LBP. The pCas9‐survivin (a typical CRISPR/Cas9 plasmid that targets and knockouts the *survivin* gene) delivered by LBP was used to evaluated the in vivo gene editing and anti‐cancer efficiencies through the nude mouse orthotopic HCC model. Additionally, we also examined if sensitization exists between pCas9‐survivin and sorafenib (SF, a widely clinically used multi‐kinases target therapy drug for HCC^[^ ^36^ ^]^) to treat orthotopic HCC in mice.

## 2 Results and Discussion

### 2.1 Preparation and Characterization of LBP

**Figure 1:**
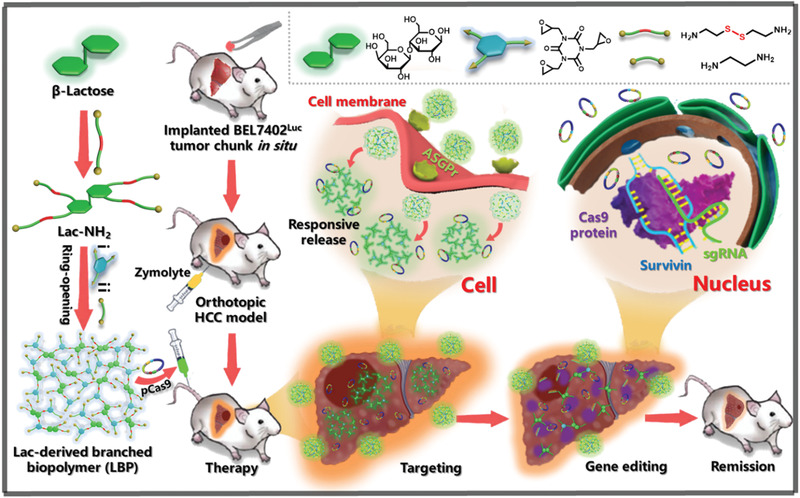
Schematic illustration of the preparation of lactose‐derived branched biopolymer (LBP) and the resultant delivery and gene editing processes with pCas9‐survivin to treat orthotopic hepatocellular carcinoma (HCC). A lactose‐derived branched cationic biopolymer (LBP) with plentiful bio‐reducible disulfide linkages and hydroxyl groups was first synthesized via a facile one‐pot ring‐opening reaction, and the LBP‐mediated delivery of pCas9‐survivin, which could target and knockout *survivin* oncogene, showed effective gene editing and anti‐cancer activities in orthotopic HCC mouse model.

The detailed synthetic route of LBP was shown in **Figure** **1**. First of all, amino groups‐modified lactose, Lac‐NH_2_, was prepared by the reaction of *β*‐lactose and cystamine (CA). Typical ^1^H NMR spectra of Lac‐NH_2_ were shown in Figure S1, Supporting Information. The two signals from *δ* = 2.5–3.4 ppm referred to the protons on the alkyl chains of CA, which proved that Lac‐NH_2_ was synthesized successfully. Based on the calculated integration, there were about 2 (or 4) amino groups on Lac‐2NH_2_ (or Lac‐4NH_2_).

Then, LBP^2^ (or LBP^4^) was synthesized via a one‐pot ring‐opening reaction of Lac‐2NH_2_ (or Lac‐4NH_2_) and triglycidyl isocyanurate (TGIC) with three epoxy groups. At the end of the reaction, excess ethylenediamine (ED) was added to consume the residual epoxy groups. Based on the gel permeation chromato‐graphy (GPC) characterizations, the number average molecular weight (*M*_n_) and polymer dispersity index (PDI) of LBP^2^ and LBP^4^ were determined and shown in Table S1, Supporting Information. LBP^2^ (≈1.8 × 10^4^ g mol^−1^) and LBP^4^ (≈2.1 × 10^4^ g mol^−1^) possess comparable molecular weights.

### 2.2 Biophysical Properties of LBP

The nucleic acid compacting ability of LBP was examined first. As shown in Figure S2a, Supporting Information, agarose gel electrophoresis assays demonstrated that LBP could efficiently compact pDNA (pRL‐CMV, a reporter plasmid encoding *luciferase* gene) within the mass ratio of 1.0–2.0. Next, the particle sizes and zeta potentials of LBP/pDNA complexes at different mass ratios were characterized. It was shown that particle sizes range from 200 to 350 nm and zeta potentials range from 20 to 40 mV (Figure S2b, Supporting Information). The particle sizes of complexes decreased with raising mass ratios and the zeta potentials increased and then became stable, which were consistent to our previous studies.^[^ ^16^, ^31^, ^33^ ^]^

**Figure 2:**
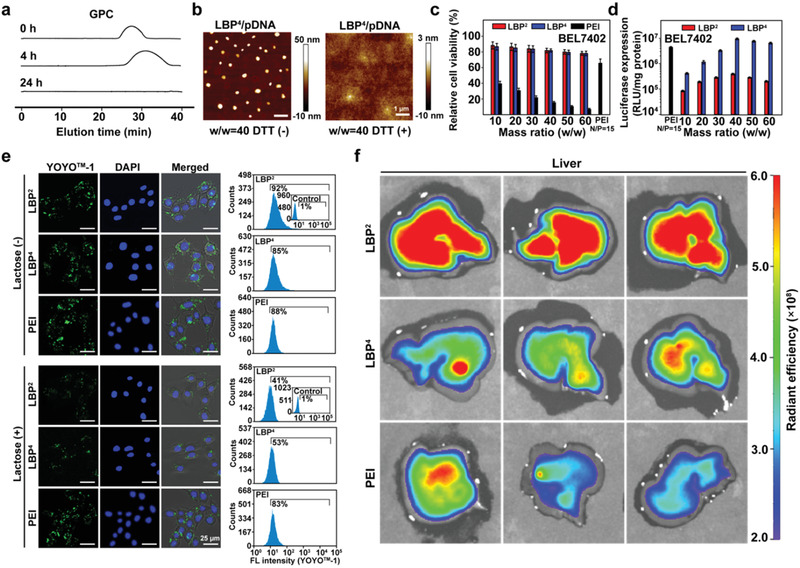
a) GPC characterizations of LBP^4^ in the presence of DTT (10 mm) at different time points. b) AFM images of LBP^4^/pDNA complex at the mass ratio of 40 in the absence (−) and presence (+) of DTT. c) Cytotoxicity of LBP/pDNA and PEI/pDNA complexes in BEL7402 cell lines at various mass ratios (mean ± SD, *n* = 3). d) In vitro gene transfection efficiencies of LBP/pDNA complexes at mass ratios from 10 to 60 in BEL7402 cell lines in comparison with those mediated by PEI (*M*_w_ ≈ 25 kDa) at its optimal N/P ratio of 15 (mean ± SD, *n* = 3). e) CLSM images and flow cytometry of BEL7402 cells treated with LBP^2^/pDNA or LBP^4^/pDNA at the mass ratio of 40 and PEI/pDNA at the N/P ratio of 15 for 4 h in the absence (−) and presence (+) of lactose, where YOYO‐1‐labeled pDNA was shown in green, and DAPI‐labeled nuclei were shown in blue. f) Representative fluorescence images of livers in different treatment groups (*n* = 3).

Biodegradable capability is another essential biophysical property for gene carriers, which could reduce the toxicity of carriers. LBP possesses rich reduction‐responsive disulfide linkages. As a result, LBP could be degraded in the presence of reducing agents. GPC assays provided direct evidences of degradable behaviors. LBP^4^ was selected as the typical polycation for the degradation assay. LBP^4^ gradually degraded in the presence of dithiothreitol (DTT) from 0 to 24 h (**Figure** **2**a). In addition, atom force microscope (AFM) imaging was also used to prove degradable behaviors of LBP^4^. In comparison with LBP^4^/pDNA complexes in normal environment, LBP^4^ could not form stable nanoparticles with pDNA in the presence of reducing agent DTT (Figure 2b). To further prove the reduction‐responsive property of LBP, the pDNA compacting ability of LBP was evaluated in the presence of DTT. The presence of DTT promoted the leakage of pDNA from LBP/pDNA complexes (Figure S2c, Supporting Information). The migration of pDNA was retarded at the higher mass ratio of 2.0–2.5. This phenomenon indicated that LBP/pDNA complexes were unstable in reducing environment. Moreover, after incubation with heparin in the presence of DTT, the rapid pDNA release from complexes almost occurred at all tested mass ratios. LBP/pDNA formulations in reducing environment could be entirely decomposed via inter‐exchange with counter polyanions. These results indicated that the reduction‐responsive LBP could become unstable and accelerate the release of nucleic acids in intra‐cellular reducing environment.

All the above biophysical characterizations demonstrated that LBP possess good nucleic acid compacting ability and reduction‐responsive degradability, which demonstrates that LBP is a potential, impressive gene vector.

### 2.3 In Vitro Characterization with Reporter Plasmids

The cytotoxicity of LBP/pDNA complexes at various mass ratios were evaluated via the thiazolyl blue tetrazolium bromide (MTT) assay in HEK293 and BEL7402 cell lines (Figure S3a, Supporting Information; Figure 2c). As the golden standard of polycationic transfection reagent, polyethylenimine (PEI, ≈25 kDa) was used as the control.^[^ ^16^, ^20^ ^]^ In comparison with PEI/pDNA complexes at the same mass ratios from 10 to 60 or its optimal N/P ratio for both cell lines, the cell viability of LBP/pDNA complexes was significantly higher. The reduction‐responsive degradability of LBP reduced the cytotoxicity, which is consistent with the earlier reports.^[^ ^20^, ^31^ ^]^ Moreover, the rich hydroxyl groups could also shield the cytotoxicity caused by positive charges.^[^ ^16^, ^20^ ^]^

In vitro transfection performances were assessed with a pRL‐CMV reporter plasmid (pDNA). First of all, the transfection efficiency of PEI/pDNA complexes at the N/P ratios from 5 to 30 in BEL7402 cells was examined. As shown in Figure S3b, Supporting Information, the optimal N/P ratio of PEI/pDNA complexes was 15 in BEL7402 cells. The subsequent transfection performances of LBP/pDNA were analyzed at mass ratios from 10 to 60 in HEK293 and BEL7402 cell lines, where PEI/pDNA at its optimal N/P ratio for both cells was taken as the contrasts (Figure S3c, Supporting Information; Figure 2d). The transfection efficiency of LBP first increased and then decreased with increasing mass ratios. The optimal mass ratio was 40–50 for both cell lines. In comparison with LBP^2^, LBP^4^ possessed more branches and exhibited the better transfection efficiency at the same mass ratio.

To further confirm the transfection performance of LBP, the delivery of another reporter plasmid pEGFP‐N1 encoding the enhanced green fluorescent protein (EGFP) gene was examined. The representative images of EGFP expressions were showed after transfection of pEGFP‐N1 using LBP^4^, LBP^2^, or PEI (Figure S4, Supporting Information). Based on the flow cytometry analysis, the percentages of the EGFP positive cells of the LBP^4^, LBP^2^, and PEI groups in HEK293 (or BEL7402) were 59% (or 25%), 28% (or 12%), and 53% (or 13%), respectively. LBP^4^‐transfected HEK293 and BEL7402 cells showed significantly more EGFP positive signals than the LBP^2^ counterpart.

### 2.4 Characterization of Targeting Ability

In addition to in vitro cytotoxicity and transfection performances, ASGPr‐mediated targeting capability of LBP was also investigated. First, two of ASGPr‐specific staining agents, MAL‐FITC and SNA‐FITC were used to characterize expression levels of ASGPr in HEK293 and BEL7402 cell lines. It was showed that ASGPr expression on the surface of BEL7402 cells was higher than that of HEK293 cells (Figure S5, Supporting Information).

Subsequently, the ASGPr‐mediated targeting performances of LBP were estimated by analyzing the efficiencies of cellular uptake of LBP/pDNA complexes in both cell lines under the absence or presence of *β*‐lactose, where pDNA was labeled with YOYO‐1 staining agent to provide detection signals for flow cytometry analyses. In BEL7402 cells (Figure 2e), the percentages of the positive cells treated with LBP^2^/pDNA and LBP^4^/pDNA were 92% and 85%. The cells treated with the LBP^2^/pDNA complexes exhibited more green aggregations than the LBP^4^/pDNA counterpart under the absence of *β*‐lactose. This may be due to the greater number of remaining groups in modified *β*‐lactose in LBP^2^ which can specifically recognize ASGPr. When LBP/pDNA complexes were investigated in BEL7402 cells in the presence of *β*‐lactose, ASGPr‐mediated endocytosis process was inhibited due to the occupation of additional *β*‐lactose. As a result, less green aggregations of the LBP^2^/pDNA and LBP^4^/pDNA complexes were observed comparing with the lactose‐free cases. The percentages of the positive cells treated with LBP^2^/pDNA and LBP^4^/pDNA in the presence of *β*‐lactose were 41% and 53%, respectively. To further illustrate the targeting ability of LBP, non‐targeting PEI was used as a negative control, and no obvious differences were observed among the percentages of the positive cells treated with PEI/pDNA in the absence and presence of *β*‐lactose. The endocytosis process in HEK293 cells (Figure S6, Supporting Information) was less inhibited than in BEL7402 cells, where there was low ASGPr expression on the surface of HEK293 cells.

The targeting property of LBP was also proved in vivo. Cy7‐NHS was used to label LBP and PEI. As shown in Figure 2f and Figure S7a, Supporting Information, after 4‐h tail vein injection, in comparison with PEI, both LBP^2^ and LBP^4^ appeared more in liver, and the degree of LBP^2^ enrichment was greater (Figure S7b, Supporting Information), which is in line with the in vitro results. This may be due to more ASGPr‐binding groups in LBP^2^ compared to LBP^4^. The transfection efficiencies of LBP^2^ and LBP^4^ in vivo were further characterized. Reporter plasmid pRL‐CMV was used here. PEI/pDNA was used as the control (Figure S7c, Supporting Information). At the 12th day after tail vein injection (administered every other day), the mice treated with LBP^4^/pDNA showed higher luciferase expression than the mice treated with LBP^2^/pDNA or PEI/pDNA.

In summary, both LBP^2^ and LBP^4^ have low cytotoxicity. Although LBP^4^ did not exhibit excellent targeting property like LBP^2^, LBP^4^ had better transfection performance than LBP^2^ in vivo and in vitro. Both targeting ability and transfection performance should be considered together for further applications. Thus, LBP^4^ with balanced targeting and transfection capabilities was used in the following parts to deliver CRISPR/Cas9 systems for gene editing and anti‐cancer assays.

### 2.5 In Vitro Transfection and Gene Editing Assays with pCas9‐Survivin

**Figure 3:**
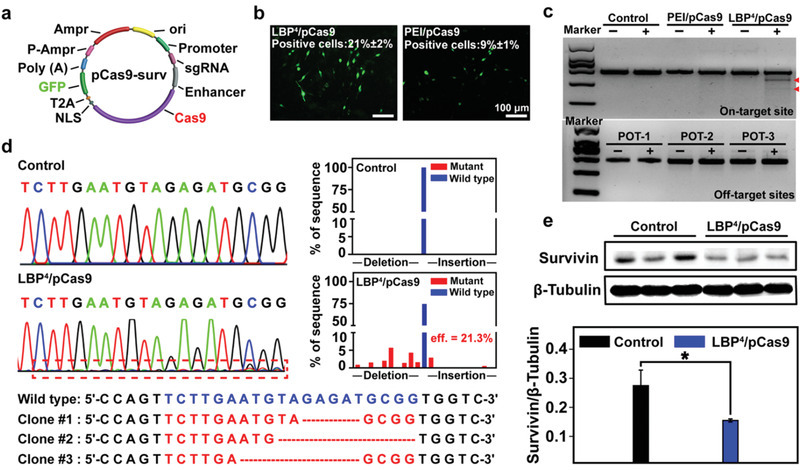
a) Structure of pCas9‐survivn. b) Representative images of GFP expression mediated by the LBP^4^/pCas9 and PEI/pCas9 complexes in BEL7402 cells at the 24th hour after transfection, and the percentages of positive cells were determined by flow cytometry analyses. c) Agarose gel electrophoreses of enzyme digestion PCR products amplified from the *survivin* locus and off‐target sites in BEL7402 cells in the absence (−) and presence (+) of T7EI enzyme. d) Sanger sequencing analysis and T‐A cloning sequencing results of PCR amplicons of the targeted sites in control and LBP^4^/pCas9 groups. e) Western blot of three transfections with LBP^4^/pCas9 and their controls, and corresponding statistical analyses of relative content of survivin protein expression in control and LBP^4^/pCas9 at the 48th hour after transfection (mean ± SD, *n* = 3, *p < 0.05).

The optimized plasmid pCas9‐survivin (pCas9) encoding *Cas9* and green fluorescent protein (GFP) as well as transcribing single guide RNA (sgRNA)^[^ ^16^ ^]^ (**Figure** **3**a) was used to be delivered into human HCC BEL7402 cells. Plasmid pCas9 could target and knockout oncogene *survivin* via the Cas9 protein under the guidance of transcribed sgRNA, which plays a pivotal role in unlimited proliferation of cancer cells and could impede apoptosis of cancer cells.^[^ ^37^, ^38^ ^]^ The loss‐of‐function editing of oncogene *survivin* inhibits the malignant proliferation of cancer cells and increases the sensitivity of cancer cells to anti‐neoplasia drugs. Sorafenib (SF) is one of target therapy drugs for treating HCC in clinic.^[^ ^36^ ^]^ Therefore, the combined use of pCas9 with sorafenib was proposed for better anti‐cancer activities.

GFP expression was used to visually assess the transfection performance of LBP^4^/pCas9 in BEL7402 cells (Figure 3b; Figure S7d, Supporting Information). After 24 h transfection, the LBP^4^ group showed much more GFP‐positive cells compared to the PEI group. Based on the flow cytometry analysis, the percentages of the GFP‐positive cells mediated by LBP^4^ and PEI were about 21% and 9%, respectively. Better transfection performance of LBP^4^ was consistent with those using the above reporter plasmids pRL‐CMV and pEGFP‐N1.

The gene‐disruption ability of the LBP^4^/pCas9 complex was investigated by the detection of enzyme mismatch cleavage and sequencing assays. As shown in the T7EI assay (Figure 3c), the cleavage bands were visible in targeted locus after cells were transfected with the LBP^4^/pCas9 complexes comparing with the negative control (PBS) group and PEI/pCas9 group. We also performed the T7EI assay on several potential off‐target sites and found no obvious cleavage bands, indicating that there were no evident off‐target effects.

Sanger DNA sequencing results showed that miscellaneous peaks appeared at the same location of PCR fragments in comparison with the negative control group (Figure 3d). The efficiency of gene editing was 21.3% for LBP^4^/pCas9. Deletion of nucleotide residues occurred most frequently in mutant amplicons at the editing site. Subsequent T‐A cloning sequencing results further verified the deletion of nucleotide residues due to CRISPR/Cas9 genome editing. These results confirmed the genome‐editing capability mediated by the LBP^4^/pCas9 complexes. We also detected survivin protein expression levels via western blot (WB) (Figure 3e). Compared to the control group, the BEL7402 cells treated with LBP^4^/pDNA showed the decreased survivin expression.

**Figure 4:**
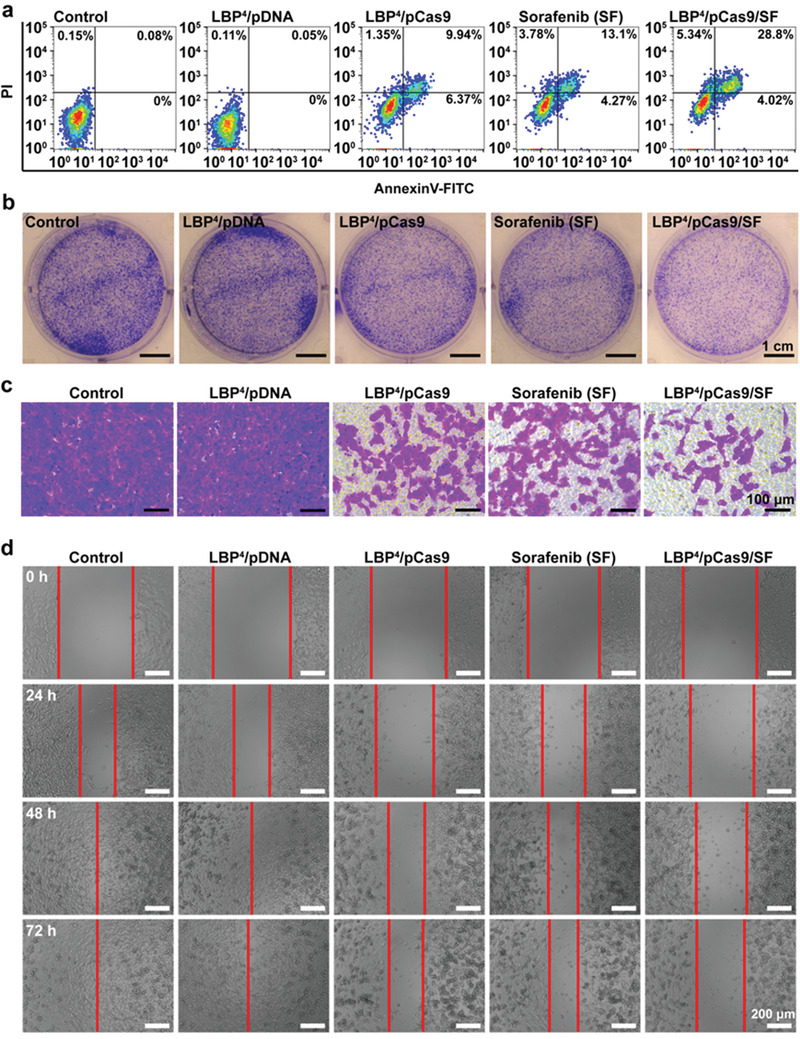
a) Flow cytometry analyses of the percentages of apoptotic cells at the 72nd hour after transfection in different treatment groups. b) Representative cloning formation images in different treatment groups. c) Representative images of BEL7402 cells in different treatment groups on the surface of lower chambers of Matrigel transwell at the 48th hour after transfection. d) Representative images of wound‐healing assays of BEL7402 cells in different treatment groups from 0 to 72 h.

Knockout oncogene *survivin* via pCas9 could significantly inhibit HCC cell proliferation via restoring their apoptosis capability (Figure S8a, Supporting Information). LBP^4^/pCas9/SF showed the best inhibitory efficacy for HCC cells, further revealing that pCas9 enhanced sensitivity of cancer cells to drugs. Subsequently, apoptosis analyses were also performed to further prove the apoptosis‐enhancing property of pCas9 (**Figure** **4**a). At 72 h after transfection, the percentages of apoptotic BEL7402 cells treated with PBS, LBP^4^/pDNA, LBP^4^/pCas9, SF, and LBP^4^/pCas9/SF were 0.08%, 0.05%, 16.31%, 17.37%, and 32.82%, respectively (Figure S8b, Supporting Information). Similarly, more apoptotic BEL7402 cells were detected in the LBP^4^/pCas9/SF group. Cloning formation assays demonstrated that the LBP^4^/pCas9 complex has less crystal violet aggregation compared with the control group or the LBP^4^/pDNA group. Among all these groups, the LBP^4^/pCas9/SF led to the least aggregation (Figure 4b).

Since survivin also play an important part in the invasion and metastasis of HCC, we then examined impacts of pCas9 on the invasion and migration capabilities of HCC cells. The impact of pCas9 on the invasiveness of BEL7402 cells was determined using Matrigel transwell assays. The reduced invasion ability of HCC cells was clearly observed after transfection with pCas9. Among all groups, LBP^4^/pCas9/SF still showed the best inhibitory effects on cell invasion (Figure 4c). Next, wound‐healing assays showed that pCas9 delivered by LBP^4^ could more effectively inhibit cell motility compared to the control group or the LBP^4^/pDNA group. In particular, LBP^4^/pCas9/SF exhibited the lowest percentage of wound closure among all groups (Figure 4d; Figure S8c, Supporting Information), indicating that knockout of the *survivin* gene increased drug sensitivity of HCC cells to sorafenib.

All these results demonstrated that the LBP^4^/pCas9 complex alone could produce efficient gene editing and obviously inhibit the malignant phenomena of HCC cells. Moreover, LBP^4^/pCas9 complex in combination with SF demonstrated the better anti‐cancer effects. These data elucidated that loss‐of‐function gene editing reactivated the programmed cell apoptosis and promoted the sensitivity of cancer cells to anti‐cancer drugs.

### 2.6 In Vivo Gene Editing for Orthotopic Human HCC Inhibition

To evaluate in vivo anti‐neoplastic effects, LBP^4^‐delivered pCas9 was used to treat mice with orthotopic HCC. HCC BEL7402 cells with stable luciferase expression (BEL7402‐Luc) were used to establish the in situ human HCC models in liver of nude mice (Figure 1). When the mouse model was successfully established through bioluminescence imaging, tumor‐bearing mice with basically uniform bioluminescence intensity were randomly divided into five groups: PBS (control), LBP^4^/pDNA, LBP^4^/pCas9, free SF, and LBP^4^/pCas9/SF. All mice were administrated with different reagents through tail vein injection.

**Figure 5:**
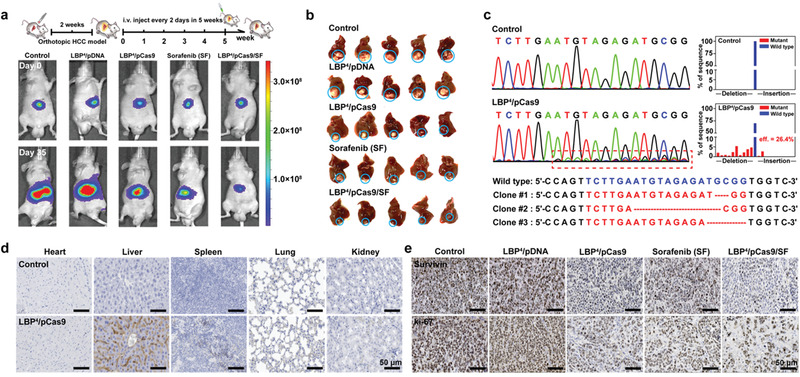
a) Representative bioluminescence, images of each treatment group at 0th and 35th day (*n* = 5). b) Liver images of each treatment group, where the orthotopic tumor nodules were highlighted with blue solid circles (*n* = 5). c) Sanger sequencing analyses and T‐A cloning sequencing results of PCR amplicons of the targeted sites in the control and LBP^4^/pCas9 groups. d) Inmmunohistochemical analyses of Cas9 protein expression in heart, liver, spleen, lung, and kidney of the control and LBP^4^/pCas9 groups. e) Inmmunohistochemical analyses of survivin and ki‐67 proteins expressions in tumor tissues after different treatments.

Tumor proliferation was evaluated using in vivo bio‐luminescence imaging (**Figure** **5**a; Figure S9a, Supporting Information) and tumor radiant efficiency (Figure S9b, Supporting Information). In comparison with the control and LBP^4^/pDNA groups, the in situ HCC growth of the LBP^4^/pCas9 and free SF groups was significantly inhibited (*p < 0.05). Interestingly, the LBP^4^/pCas9/SF group showed the most evidently inhibitory effect on orthotopic HCC in mice. These results were in line with the above in vitro data. The body weights of mice in each group were monitored during the period of treatments (Figure S9c, Supporting Information). No significant body weight loss was observed, which demonstrated that LBP has no potential toxicity in vivo. At the 35th day of treatments, all mice were sacrificed. The livers were removed and imaged (Figure 5b). White tumor nodules on the surface of livers were highlighted with blue solid circles. The tumors of the LBP^4^/pCas9/SF group had the minimal volume among all five groups, which was consistent with the in vivo bioluminescence imaging. These results confirmed that the combination of gene therapy and chemotherapy had the most significant tumor inhibition activity than single therapy mode.

In vivo gene editing efficiency of the LBP^4^/pCas9 complex was examined by Sanger sequencing. The genomic DNA was extracted from tumor tissues of the control and LBP^4^/pCas9 groups. In comparison with the control group, miscellaneous peaks appeared at the same position of *survivin* in in situ tumors of the LBP^4^/pCas9 group, indicating that *survivin* was gene‐edited by pCas9 in vivo. It was calculated that the gene editing efficiency was 26.4% in orthotopic HCC. T‐A cloning of the PCR products plus Sanger sequencing elucidated that nucleotides deletion occurred most frequently at the gene editing sites (Figure 5c).

Expression levels of Cas9 protein in the tumors of different groups were then examined via immunohistochemical (IHC) analyses. Figure 5d showed that there were more Cas9 proteins expressed in the LBP^4^/pCas9 group compared with the control group. The expressions of survivin and ki‐67 proteins were also evaluated (Figure 5e). ki‐67 was a well‐known tumor proliferation marker, which is commonly overexpressed in tumor tissues.^[^ ^39^ ^]^ It was found that evidently decreased survivin and ki‐67 expressions exist in the tumors of the LBP^4^/pDNA group. IHC assays showed that LBP^4^/pCas9/SF induced most significantly reduced ki‐67 protein expression in orthotopic HCC tissues. In addition, the H&E stainings of the major mouse organs, such as heart, liver, spleen, lung, and kidney, indicated that there were no significant changes of organs after the mice received different treatments (Figure S10, Supporting Information).

All the above orthotopic human HCC assays confirmed that through the efficient loss‐of function oncogene editing in vivo, pCas9 delivered by LBP^4^ has an effective cancer inhibitory property and can promote the sensitivity of drug.

## 3 Conclusion

A lactose‐derived biopolymer (LBP) was successfully prepared by one‐pot ring‐opening reactions as a safe CRISPR/Cas9 delivery system for efficient genome editing in vivo to treat orthotopic hepatocellular carcinoma (HCC). Based on the biophysical and cytological characterizations, LBP possesses good reduction‐responsive degradability, biocompatibility, transfection performances, and ASGPr‐mediated targeting capability. As a typical CRISPR/Cas9 system, pCas9‐survivin was explored to inhibit viability of HCC. The LBP‐mediated delivery of pCas9‐survivin demonstrated efficient gene editing in vitro, made HCC cells apoptosis and inhibited proliferation. Consistently, in vivo biodistribution and anti‐orthotopic tumor assays further confirmed that the effective loss‐of‐function oncogene editing via liver‐targeting LBP‐mediated delivery of CRISPR/Cas9 system was safe and applicable. In addition, the LBP/pCas9 complex also significantly enhanced anti‐neoplasia effects of drugs through inducing oncogene *survivin* knock‐out. The current study developed a safe approach for efficient genome editing of CRISPR/Cas9 system in vitro and in vivo.

## 4 Experimental Section

##### Materials and Cells

*β*‐Lactose (99%), *N*, *N′*‐carbonyldiimidazole (CDI, 98%), cystamine dihydrochloride (CA 2HCl, 98%), 1, 3, 5‐triglycidyl isocyanurate (TGIC, 98%), ethylenediamine (ED, 98%), triethylamine (TEA, 99.5%), branched polyethyleneimine (PEI, 98%, *M*_w_ ≈ 25 kDa, the golden standard of nonviral gene vectors), dithiothreitol (DTT, 99%), deuterium oxide (D_2_O, 99%), and methylthiazolyldiphenyl‐tetrazolium bromide (MTT, 98%) were obtained from Energy & Chemical Co., Ltd. (Shanghai, China). Sorafenib (SF, 99%) was obtained from AMQUAR Biological Technology Co. (Shanghai, China). ASGPr‐specific staining agents of MAL‐FITC and SNA‐FITC were purchased from VECTOR Co., Ltd., USA. HEK293 and BEL7402 cellular lines were provided by Beijing Beina Science & Technology Co., Ltd. Renilla Luciferase Assay Kit (Promega, 1000 assays), dulbecco's modified eagle medium (DMEM), fetal serum bovine (FBS), penicillin‐streptomycin solution (P/S, penicillin: 1 × 10^4^ units mL^−1^, streptomycin: 10 mg mL^−1^), 4′,6‐diamidino‐2‐phenylindole (DAPI, 1 mg mL^−1^ solution in deionized water), YOYO‐1 staining reagent (1 × 10^−3^ mol L^−1^ in DMSO), AnnexinV‐FITC/PI Apoptosis Kit (20 assays), Cyanine7‐*N*‐hydroxysuccinimide ester (Cy7‐NHS, 1 mg), lgG‐HRP (10 mg mL^−1^), and Protein Extracted Kit (50 assays) were obtained from Beijing Solarbio Science & Technology Co., Ltd., China. Plasmid pCas9‐survivin (encoding *Cas9* and *GFP* as well as transcribing *survivin* sgRNA 5′‐TCTTGAATGTAGAGATGCGG‐3′') and qTR‐PCR primers for detecting *survivin* expression (5′‐CGCCATTAACCGCCAGATTT‐3′ and 5′‐AACAAAGCCCATCGAGGCTG‐3′) were obtained from GenScript Biotechnology Co., Ltd. (Nanjing, China). Plasmids pCas9‐survivin and other reporter plasmids (pRL‐CMV (encoding renilla luciferase), pEGFP‐N1 (encoding EGFP)) were amplified in *Escherichia coli*. Taq PCR MasterMix (2×) and T7 Endonuclease I (T7EI) were obtained from Cell Biolabs, Inc., USA. Anti‐survivin antibody and anti‐ki‐67 antibody were purchased from Abcam Co., Ltd. (USA). Nude BALB/c mice (6‐week‐old) were purchased from Beijing HFK Bioscience Co., Ltd., China.

##### Synthetic Procedure and Characterizations

First, 513 mg of *β*‐lactose and 500 mg or 1 g of CDI were added into a 50 mL‐flask containing 10 mL of anhydrous DMSO, and the reaction was conducted at room temperature for 24 h under a nitrogen atmosphere. When the reaction was finished, 5 mL of anhydrous DMSO including 4 or 10 g of de‐protected CA 2HCl was added by a 20 mL‐injection syringe. The reaction was also conducted at room temperature for 24 h. When the reaction was finished, 150 mL of acetone was used to precipitate the crude products. After washed with excess acetone for several times, white powders were obtained during the low‐speed centrifugation and vacuum drying. According to the ^1^H NMR spectra recorded on a Bruker ARX 400 MHz spectrometer (German) with D_2_O as the solvents (*δ* = 4.8 ppm), the products were named as Lac‐2NH_2_ with about two amino groups or Lac‐4NH_2_ with about four amino groups. Second, LBP was synthesized by the one‐pot ring‐opening polymerizations of Lac‐NH_2_ and TGIC. 349 mg of Lac‐2NH_2_ or 527 mg of Lac‐4NH_2_ and 100 or 200 mg of TGIC were added into a 50 mL‐flask containing 5 mL of DMSO, the polymerization was conducted at 40 °C for 24 h under a nitrogen atmosphere. Then, 1 mL of ED was added to completely consume the residual epoxy groups, where the reaction was conducted at 60 °C for 3 h. Finally, the crude products were purified by using a dialysis membrane (MWCO 3500) prior to lyophilization. The corresponding products were denoted by LBP^2^ and LBP^4^. The number average molecular weight (*M*_n_) and PDI were characterized by Waters GPC system (UK) with DMSO as the eluent at a low flow rate of 1.0 mL min^−1^ at 25 °C, where branched pullulan standards were used to generate the calibration curve.

##### Biophysical Characterizations of LBPs

Reporter plasmid pRL‐CMV (pDNA, 0.1 mg mL^−1^) was used in biophysical characterizations of LBP. The processes of agarose gel electrophoresis and AFM assays were available in Supporting Information.

##### Cell Viability Assay

Reporter plasmid pRL‐CMV (pDNA, 0.1 mg mL^−1^) was used in cell viability assays of LBPs. The detailed process of cell viability was available in Supporting Information.

##### In Vitro Transfection Assay with Reporter Plasmids

Reporter plasmids pRL‐CMV (pDNA, 0.1 mg mL^−1^) and pEGFP‐N1 (0.1 mg mL^−1^) were used in in vitro transfection assays of LBPs. The detailed processes of transfection assays were available in Supporting Information.

##### Cellular Internalization

First of all, two of ASGPR‐specific staining agents, MAL‐FITC and SNA‐FITC (VECTOR, USA) were used to characterize the expressions of ASGPR on the surfaces of HEK293 and BEL7402 cell lines. For subsequent cellular uptake assays of the LBP/pDNA complexes, pDNA was labeled with YOYO‐1 staining reagent first. A total of 2 × 10^5^ HEK293 or BEL7402 cells were seeded per 20 mm cell culture dish and incubated for 24 h. Then, 50 µL of the LBP^2^/pDNA or LBP^4^/pDNA complexes (containing 2.5 µg of YOYO‐1 labeled pDNA) at the optimal mass ratio of 40 were added to each culture dish and incubated for 4 h. The culture cells were washed with PBS and the nuclei were stained with DAPI (1 mg mL^−1^) for 10 min. Finally, confocal laser scanning microscope (CLSM made in German, Leica SP8) was used to visualize the cells and flow cytometry (FCM, Beckman Coulter, USA) was used to quantify the cellular phagocytosis efficiency. In order to prove the ASGPR‐mediated targeting ability of LBPs, 100 µL of *β*‐lactose solution (2 mg mL^−1^) was added to culture medium for HEK293 and BEL7402 cell lines and incubated for 4 h prior to the transfection with the LBP^2^/pDNA or LBP^4^/pDNA complexes. Then, the original culture medium was replaced with fresh culture medium. The LBP^2^/pDNA or LBP^4^/pDNA complex was added to both cell lines under the same procedures as mentioned above. CLSM and flow cytometry were also used to analyze the cellular uptake behaviors.

##### Biodistribution Assay

In this work, all animal studies were approved by the Ethical Committee of Shandong Cancer Hospital and Institute. LBP^2^, LBP^4^, and PEI were labeled with Cy7. Nine six‐week‐old male BALB/c nude mice were then divided into three groups (*n* = 3 per group). The LBP^2^/pDNA (containing 20 µg of pDNA, w/w = 15), LBP^4^/pDNA (containing 20 µg of pDNA, w/w = 15), and PEI/pDNA (containing 20 µg of pDNA, N/P = 10) complexes were injected to the nude mice of each group through tail vein. At fourth hour after injection, nude mice were sacrificed. Their main organs (including heart, liver, spleen lung, and kidney) were removed, and the fluorescence distribution was observed and imaged via IVIS Spectrum (PerkinElmer, USA).

##### In Vivo Transfection Assay with Reporter Plasmid

Plasmid pRL‐CMV (pDNA) was used to characterize the transfection performances in vivo. In details, nine six‐week‐old male BALB/c nude mice were divided into three groups (*n* = 3 per group). The LBP^2^/pDNA (containing 20 µg of pDNA, w/w = 15), LBP^4^/pDNA (containing 20 µg of pDNA, w/w = 15), and PEI/pDNA (containing 20 µg of pDNA, N/P = 10) complexes were injected to nude mice of each group through tail vein every other days. After 12 days, the nude mice were sacrificed. Their main organs (including heart, liver, spleen lung, and kidney) were removed and 2 mm squares of tissues were obtained from each organ. 100 µL of lysate was added and the tissues were homolyzed with a tissue homogenizer (KZ‐II, Servicebio, China). Luciferase gene expression was quantified with a commercial kit (Promega, USA) using a luminometer (Lumat LB 9507, Berthhold Technologies, USA) as relative light units (RLUs) per milligram of organ tissues (RLU mg^−1^ organ), calculation method referred to the previous work of the authors.^[^ ^40^ ^]^

##### In Vitro Transfection Assay with pCas9‐Survivin

Plasmid pCas9‐survivin (pCas9) was prepared at a concentration of 0.1 mg mL^−1^ for in vitro transfection assays. To evaluate the potential transfection performances of LBPs for the CRISPR/Cas9 system, in vitro characterizations were first carried out by using plasmid pCas9 at the mass ratio of 40 in BEL7402 cells. BEL7402 cells were seeded in a 24‐well plate at 8 × 10^4^ cells per well. After 24 h incubation, 20 µL of the LBP^4^/pCas9 complex at the mass ratio of 40 (containing 1 µg of pCas9) were added to each well. The PEI/pCas9 complex at its optimal N/P ratio of 15 in BEL7402 cells were used as contrast. After 4 h, 500 µL of new medium was used to replace the original medium. After additional 20 h incubation, the transfected cells were imaged using a Leica DMIL 3000B Fluorescence Microscope. The percentage of the GFP‐positive cells was determined by a flow cytometry (Beckman Coulter, USA).

To investigate the gene disruption ability of the LBP^4^/pCas9 complex, BEL7402 cells were seeded in a 6‐well plate at 2 × 10^5^ per well. After 24 h incubation, which were treated with 80 µL of PBS or the LBP^4^/pCas9 complex at the mass ratio of 40 (containing 4 µg of pCas9). After 48 h, the transfected cells were collected and GFP‐positive cells were sorted by a flow cytometry (Beckman Coulter, USA). Genomic DNA was then extracted from sorted cells. Genomic regions flanking the target sites were amplified by PCR with the specific primers (surviving‐F and surviving‐R). The PCR products were cloned to a T‐A vector and detected by Sanger sequencing. The editing efficiency was calculated with an online tool: TIDE: Tracking of Indels by Decomposition (https://tide.nki.nl/). Meanwhile, 20 µL of amplicon was annealed by PCR. Subsequently, 1 µL of T7EI was added to the annealed PCR products and incubated at 37 °C for 50 min. Products were finally analyzed on 2% agarose gels and imaged with a Gel Doc gel imaging system (Bio‐Rad, USA). The most potential off‐target sites that are corresponding to the on‐target genome locus were identified with an online tool: Cas‐OFFinder (http://www.rgenome.net/cas-offinder/). All the off‐target sites and primers were listed in Tables S2 and S3, Supporting Information. Off target analysis procedure was similar to on‐target examination through the T7E1 assay.

Western blot was used to assess the survivin protein expression. At the 48th hour after transfection, both proteins from BEL7402 cells were extracted by Protein Extracted Kit. A total of 10 µL of protein extracts (10 mg mL^−1^) were separated on 10% sodium dodecyl sulfate polyacrylamide gel electrophoresis (SDS‐PAGE) and transferred to nitrocellulose membrane. The expression levels of survivin were determined by anti‐survivin antibody (diluted at 1:1000, Abcam, USA). The *β*‐Tubulin was used as the loading control and its expression was measured using a monoclonal anti‐*β*‐Tubulin antibody (diluted at 1:1000, Abcam, USA).

For the cell counting assay, a total of 2 × 10^5^ BEL7402 cells per well were seeded in a 6‐well plate. After 24 h incubation, 80 µL of PBS, LBP^4^/pDNA, LBP^4^/pCas9 (containing 4 µg of pCas9), sorafenib (SF, 8 µg), and LBP^4^/pCas9/SF (containing 4 µg of pCas9 and 8 µg of SF) were then added to each well, respectively. Cells were harvested by trypsin digestion at 24, 48, and 72 h, and counted via optical microscopy (Leica DFC425 C, Germany).

For the apoptosis assay, 2 × 10^5^ BEL7402 cells per well were seeded into a 6‐well plate and treated with 80 µL of PBS, LBP^4^/pDNA, LBP^4^/pCas9 (containing 4 µg of pCas9), sorafenib (SF, 8 µg), and LBP^4^/pCas9/SF (containing 4 µg of pCas9 and 8 µg of SF). After 72 h incubation, the apoptosis percentage of the above treated cells was measured by using an Annexin V‐FITC/PI Apoptosis Kit and a flow cytometry (Beckman Coulter, USA).

For the cloning formation assay, a total of 1 × 10^3^ BEL7402 cells per well were seeded into a 6‐well plate and treated with 80 µL of PBS, LBP^4^/pDNA, LBP^4^/pCas9 (containing 4 µg of pCas9), sorafenib (SF, 8 µg), and LBP^4^/pCas9/SF (containing 4 µg of pCas9 and 8 µg of SF). At the tenth day after transfection, the cells were washed with cold PBS twice and fixed with formaldehyde solution (3.7%). HCC cells were dyed with crystal violet solution (0.2%) and imaged.

For the transwell assay, the transwell chambers were coated with 100 µL of BD Matrigel overnight at 37 °C in a culture incubator containing 5% CO_2_. A total of 1 × 10^4^ transfected BEL7402 cells (in 200 µL of medium with 0.2% BSA) were added to the upper layer of transwell chambers (pore 8 µm, Corning). A medium containing 10% FBS (600 µL) was added to the lower wells. After 48 h incubation, the cells were fixed and stained. The nonmigratory cells were scraped from the upper layer of the filter with cotton swabs. The HCC cells at the lower layer of the filters through pores were stained with 0.2% crystal violet solution and imaged.

Wound healing assays were performed as described in the authors’ previous study.^[^ ^16^ ^]^ BEL7402 cells were seeded in a 12‐well plate at a density of 1 × 10^5^ cells per well in 1 mL of medium and incubated for 24 h. Then, HCC cells were treated with 40 µL of PBS, LBP^4^/pDNA, LBP^4^/pCas9 (containing 2 µg of pCas9), sorafenib (SF, 4 µg), and LBP^4^/pCas9/SF (containing 2 µg of pCas9 and 4 µg of SF. When reaching about 90% confluence, the cell layers were scratched with pipet tips. BEL7402 cells were then continued to be cultured and wound closure was quantified at 24, 48, and 72 h.

##### In Vivo Tumor Inhibition Assay

To evaluate orthotopic HCC inhibitory activities of pCas9 delivered by LBP^4^, BEL7402 orthotopic xenografts in six‐week‐old female nude BALB/c mice were established. In details, a total of 1 × 10^7^ BEL7402 cells with stable *luciferase* transfection (BEL7402‐luci) were first inoculated subcutaneously into the right rib per nude mouse. When the tumors reached 40–50 mm^3^, they were cut into small pieces of 4–8 mm^3^. Tumor masses were then implanted into the liver parenchyma of nude mice. Wound was sutured and disinfected. After two weeks, the formation of orthotopic liver tumor was detected by bioluminescence imaging (PerkinElmer, USA). The mice were anesthetized with isoflurane and inoculated intraperitoneally with substrate (concentration: 15 mg mL^−1^; dose: 0.2 mg per 20 g^−1^) prior to the bioluminescence imaging assay. When tumor models were successfully established by the confirmation of bioluminescence imaging, tumor‐bearing mice with basically uniform bioluminescence intensity were randomly divided into five groups (*n* = 5 per group) for tumor inhibitory activities: 150 µL of PBS, LBP^4^/pDNA (containing 20 µg of pDNA, w/w = 15), LBP^4^/pCas9 (containing 20 µg of pCas9, w/w = 15), sorafenib (SF, 40 mg kg^−1^), and LBP^4^/pCas9/SF (containing 20 µg of pCas9‐surv, w/w = 15; 40 mg kg^−1^ of SF) were given to each mouse of five groups for every 2 days. Here, low dosage of LBP was used to confirm the utility of LBP for in vivo biomedical applications. PBS, LBP^4^/pDNA, and LBP^4^/pCas9 were administrated by tail vein injection. Sorafenib (SF) was given to mice by mouth. Bioluminescence imaging test was performed every week to monitor the growth of orthotopic live tumors in each mouse. After 35 days, all mice were sacrificed, tumors were harvested and imaged. The main organs including heart, liver, spleen, lung, and kidney were also harvested and kept in 4% formaldehyde solution.

The orthotopic tumors and organs were sliced for the histological analyses. For IHC analyses, anti‐survivin and anti‐ki‐67 monoclonal antibodies (diluted at 1:1000, Abcam, USA) were, respectively, used to detect survivin and ki‐67 expression in HCC tissues. Hematoxylin and eosin were used as staining agents to examine in vivo toxicity. A portion of tumor tissues were separated into single‐cell suspension. After GFP‐positive cells were sorted, their genomic DNA was extracted. After PCR amplification, *survivin* gene was sequenced. The editing efficiency was calculated with an online tool: TIDE: Tracking of Indels by Decomposition.

##### Statistics Analyses

The detailed methods for statistics analyses were available in Supporting Information.

## Conflict of Interest

The authors declare no conflict of interest.

## Supporting information

Supporting Information
