# Supplementary material for: A Lactose‐Derived CRISPR/Cas9 Delivery System for Efficient Genome Editing In Vivo to Treat Orthotopic Hepatocellular Carcinoma
Source: Adv Sci (Weinh). 2020 Jul 21;7(17):2001424. doi: 10.1002/advs.202001424 (PMC7507475; doi:10.1002/advs.202001424)
Supplement: Supplementary file 1 — Supporting Information [file ADVS-7-2001424-s001.pdf]

Supporting Information

A Lactose-Derived CRISPR/Cas9 Delivery System for Efficient Genome Editing In Vivo to Treat Orthotopic Hepatocellular Carcinoma

Yu Qi, Yanli Liu, Bingran Yu, Yang Hu, Nasha Zhang, Yan Zheng, Ming Yang, and Fu-Jian Xu**

Supporting Information includes:
Experimental Section
Figure S1-S12
Table S1-S3

Experimental Section

Biophysical Characterizations of LBPs: Reporter plasmid pRL-CMV (pDNA, 0.1 mg·mL^{-1}) was used in bio-physical characterizations of LBP. All LBP/pDNA complexes were formed by mixing equal volumes of LBPs with pDNA solutions at the desired mass ratio. Agarose gel electrophoresis assay was used to test pDNA condensation ability. Particle sizes and zeta potentials of LBP/pDNA complexes were determined with a Zetasizer Nano ZS (Malvern Instruments, Southborough, MA). For the *in vitro* degradation study, LBP[4] were characterized by GPC and Atomic Force Microscopy imaging (AFM system with a Nanoscope IIIa controller, Bruker, Santa Barbara, CA) tests in the presence of DTT. Agarose gel electrophoresis was also used to investigate the degradable properties of reduction-responsive polycations in the absence and presence of DTT and heparin.

Cell Viability Assay: Reporter plasmid pRL-CMV (pDNA, 0.1 mg·mL^{-1}) was used in cell viability assays of LBPs. For the cytotoxicity evaluation of LBPs, HEK293 and BEL7402 human hepatoma cell lines were used. Both cells were seeded in 96-well plates at 1 × 10^4 cells per well for 24 h in DMEM within 10% of FBS and 1% of P/S solution. The incubation environment requires a 5% CO_2 atmosphere with 95% relative humidity at 37°C. The cytotoxicity of LBP/pDNA complexes at mass ratios of 10-60 was evaluated through the MTT assays.

In Vitro Transfection Assay with Reporter Plasmids: Reporter plasmids pRL-CMV (pDNA, 0.1 mg·mL^{-1}) and pEGFP-N1 (0.1 mg·mL^{-1}) were used in *in vitro* transfection

assays of LBPs. First of all, the transfection efficiency of PEI/pDNA complexes at the N/P ratios of 5-30 was estimated in BEL7402 cell lines. Then, the transfection performances of LBPs were investigated in HEK293 and BEL7402 cell lines using reporter plasmid pRL-CMV. Both cell lines were seeded in 24-well plates at 6×10^4 cells per well and incubated in 500 μL of DMEM within 10% of FBS and 1% of P/S solution for 24 h. Subsequently, 20 μL of LBP/pDNA complexes (containing 1.0 μg of pDNA) at mass ratios of 10-60 were added to each well. After 4 h, 500 μL of new medium was added to replace original medium. After 20 h, the cultured cells were washed twice with PBS and lysed in 70 μL of cell-culture lysis buffer reagent (Promega, USA). Luciferase gene expression was quantified with a commercial kit (Promega, USA) using a luminometer (Lumat LB 9507, Berthhold Technologies, USA) as relative light units (RLUs) per milligram of cell protein lysate ($RLU \cdot mg^{-1}$ protein), where the transfection efficiencies of PEI/pDNA complexes at its optimal N/P ratio in both cells were used as the contrasts. In addition, transfection efficiency was also assessed using plasmid pEGFP-N1 as another reporter plasmid at the mass ratios of 40 for HEK293 cells and BEL7402 cells of LBP/pEGFP-N1 complexes (containing 1.0 μg of pEGFP-N1) under the similar procedures as mentioned above. The PEI/pEGFP-N1 complex at its optimal N/P ratio of 15 in both cells was used as contrasts. The transfected cells were imaged using a Leica DMIL 3000B Fluorescence Microscope. The percentage of EGFP-positive cells was determined by a flow cytometry (FCM, Beckman Coulter, USA).

Statistics Analyses: The differences between two groups were calculated by using Student's *t* test. One-way analysis of variance was used to compare differences between three or more groups. Excel (Microsoft 2019) was used. A $^{*}P < 0.05$ was used as the criterion of statistical significance.

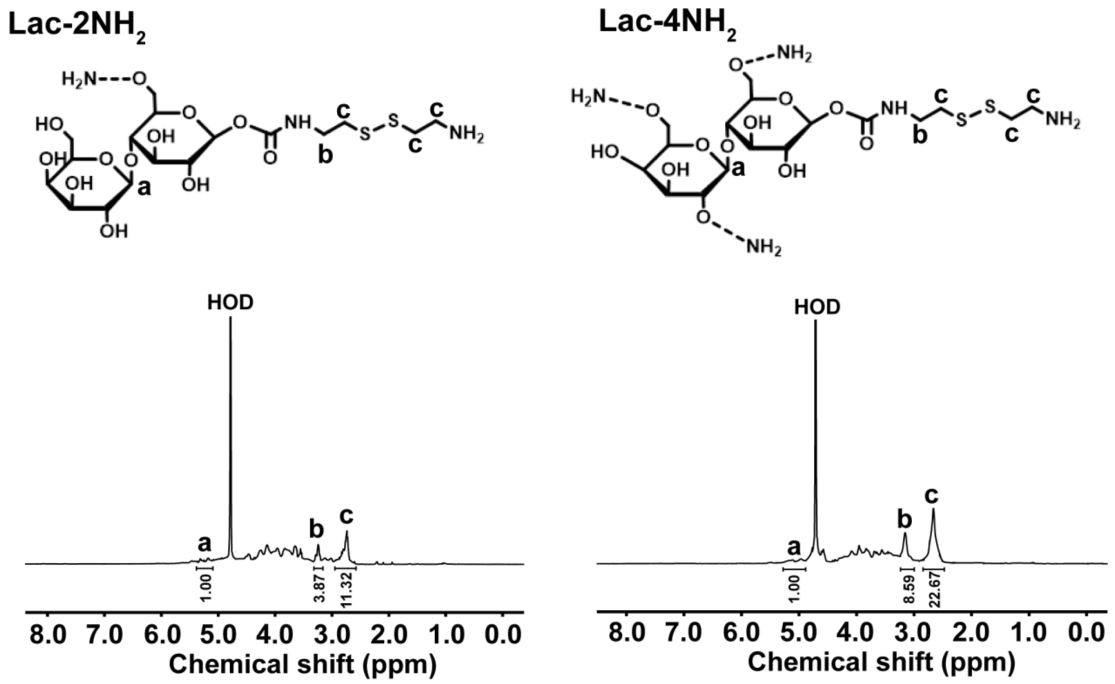

Figure S1. ^{1}H NMR spectra of Lac-NH_2 monomers

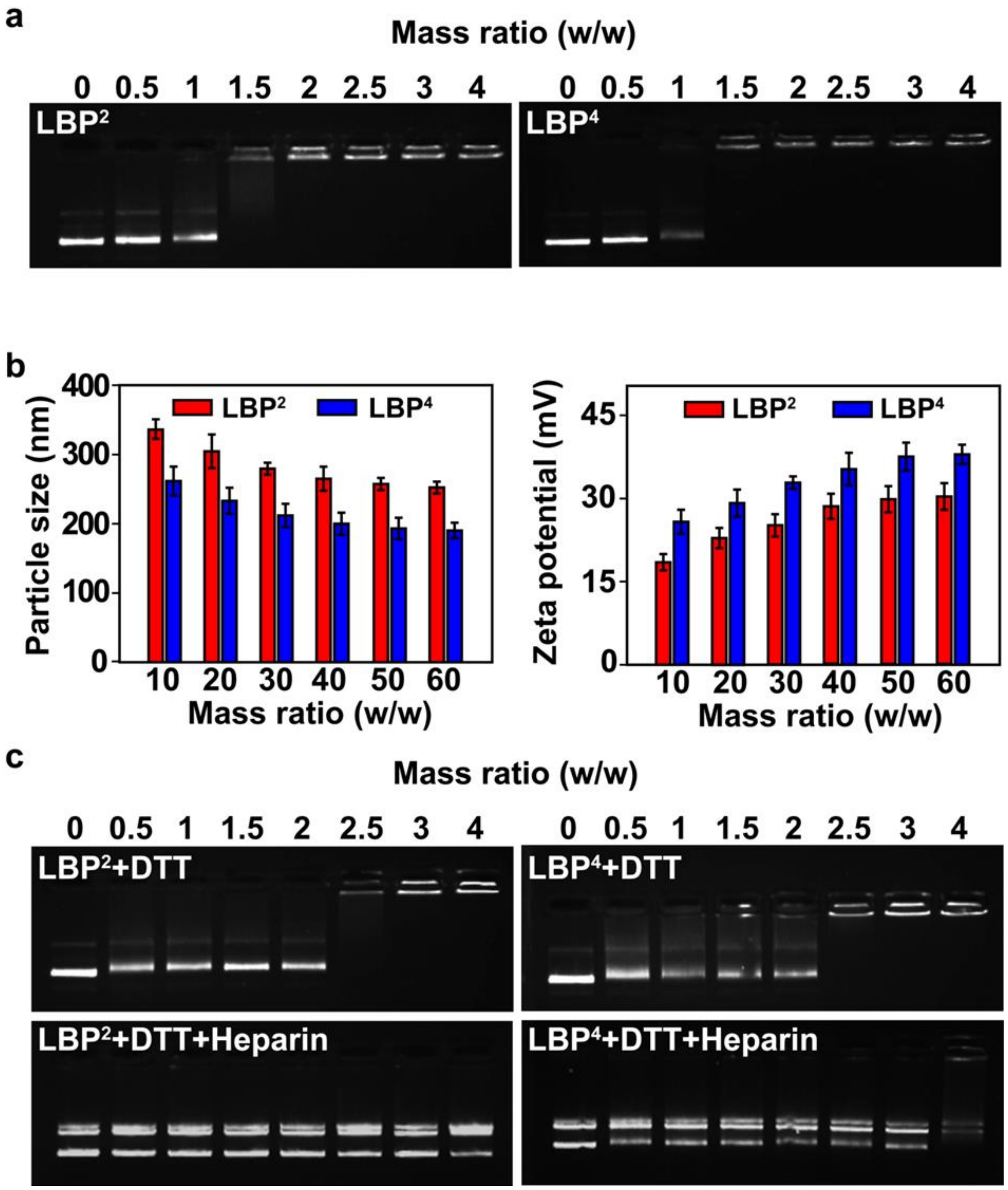

Figure S2. a) Electrophoretic mobility retardation assays of the LBP/pDNA complexes at mass ratios from 0 to 4. b) Particle sizes and zeta potentials of LBP/pDNA complexes at various mass ratios (mean ± SD, n = 3). c) Electrophoretic mobility retardation assays of responsive degradability of LBP in the presence of DTT and DTT with heparin.

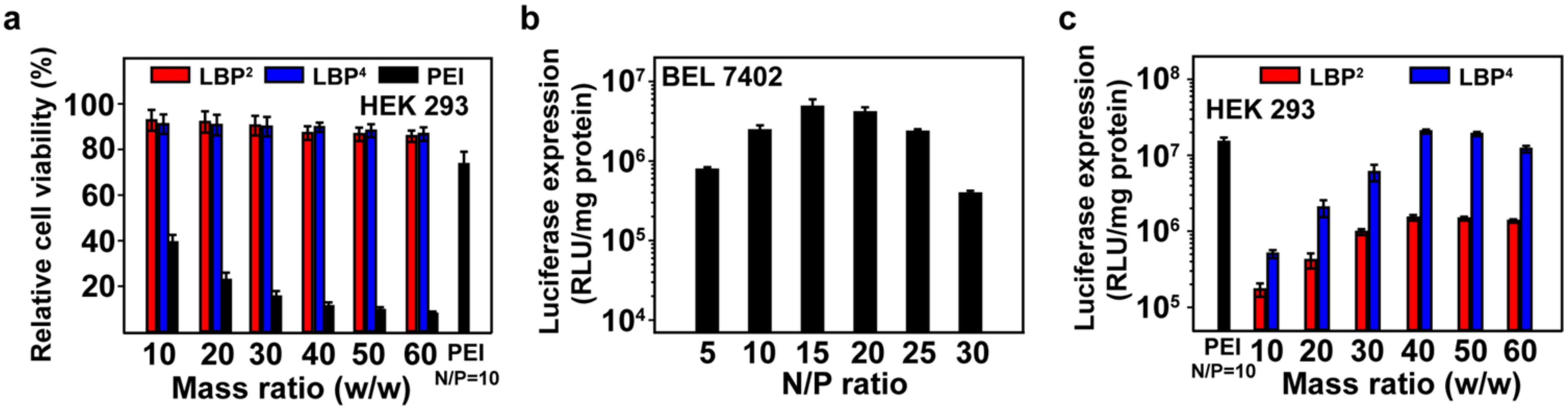

Figure S3. a) Cytotoxicity of LBP/pDNA and PEI/pDNA complexes in HEK293 cell lines at various mass ratios (mean ± SD, n = 3). b) Transfection efficiency of reporter plasmid pRL-CMV mediated by PEI ($M_w \approx 25$ kDa, golden standard) in BEL 7402 cells at the N/P ratios from 5 to 30 (mean ± SD, n = 3). c) *In vitro* gene transfection efficiencies of LBP/pDNA complexes at mass ratios from 10 to 60 in HEK293 cell lines in comparison with those mediated by PEI ($M_w \approx 25$ kDa, golden standard) at its optimal N/P ratio of 10 (mean ± SD, n = 3).

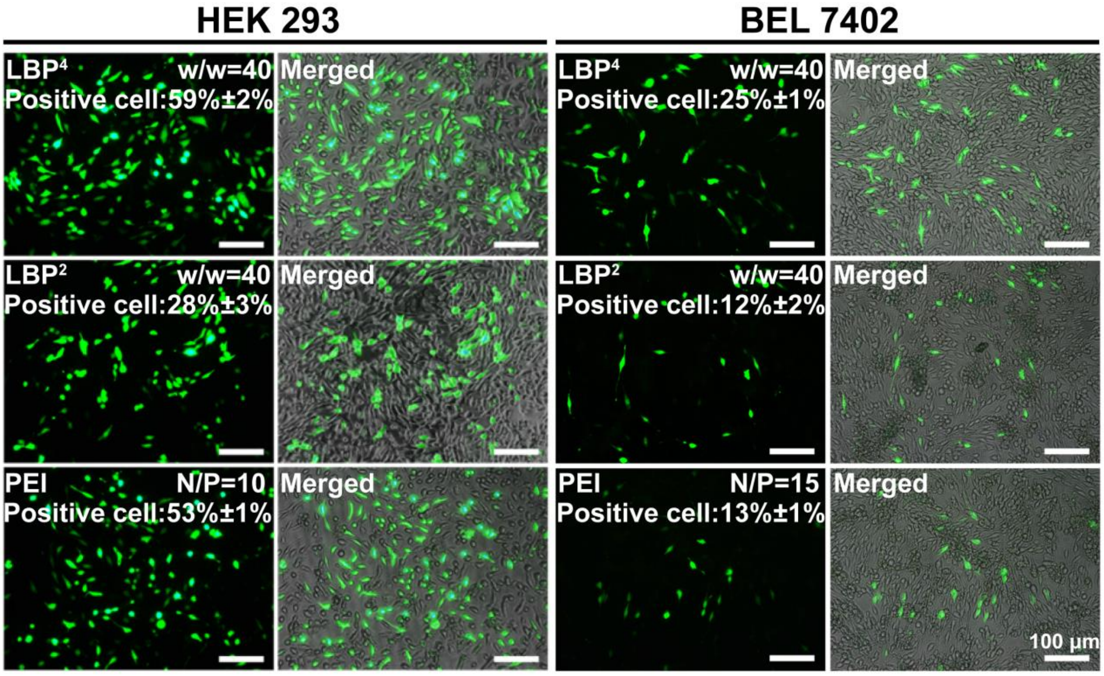

Figure S4. Representative fluorescent and merged images of EGFP expression mediated by LBP^4, LBP^2 and PEI in HEK293 and BEL7402 cell lines. The percentages of positive cells were determined by flow cytometry analyses.

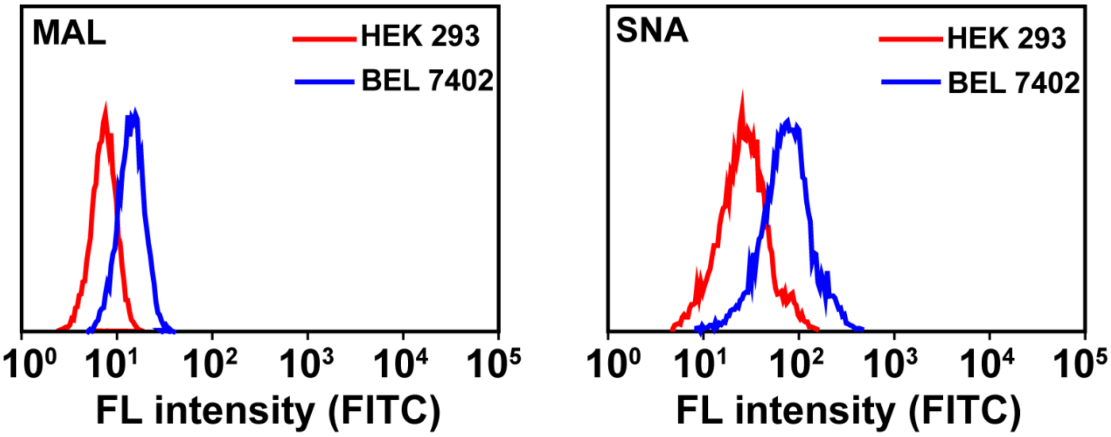

Figure S5. ASGPr expressions of HEK293 and BEL7402 cell lines were determined by MAL-FITC and SNA-FITC staining agents.

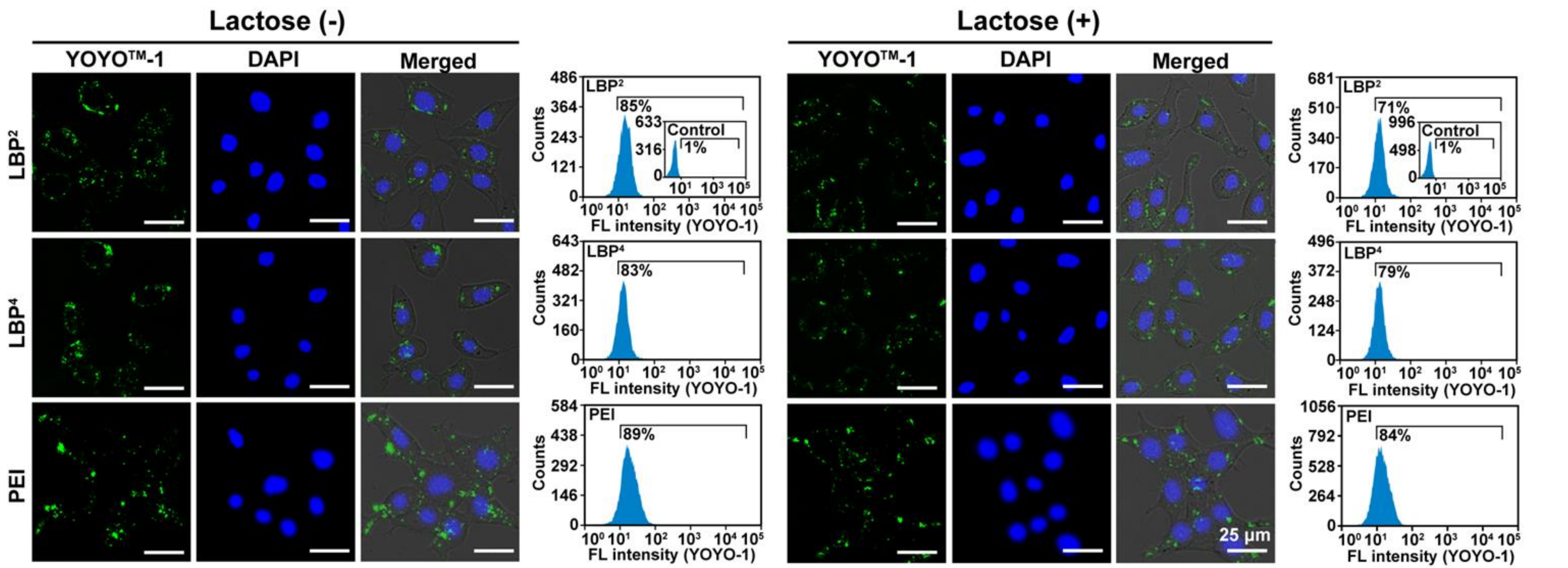

Figure S6. CLSM images and flow cytometry results of HEK293 cells treated with LBP^2/pDNA, LBP^4/pDNA at the mass ratios of 40 and PEI/pDNA at the N/P ratio of 10 for 4 h in the absence (-) and presence (+) of lactose, where YOYO^{TM}-1-labeled pDNA were shown in green, DAPI-labeled nuclei were shown in blue.

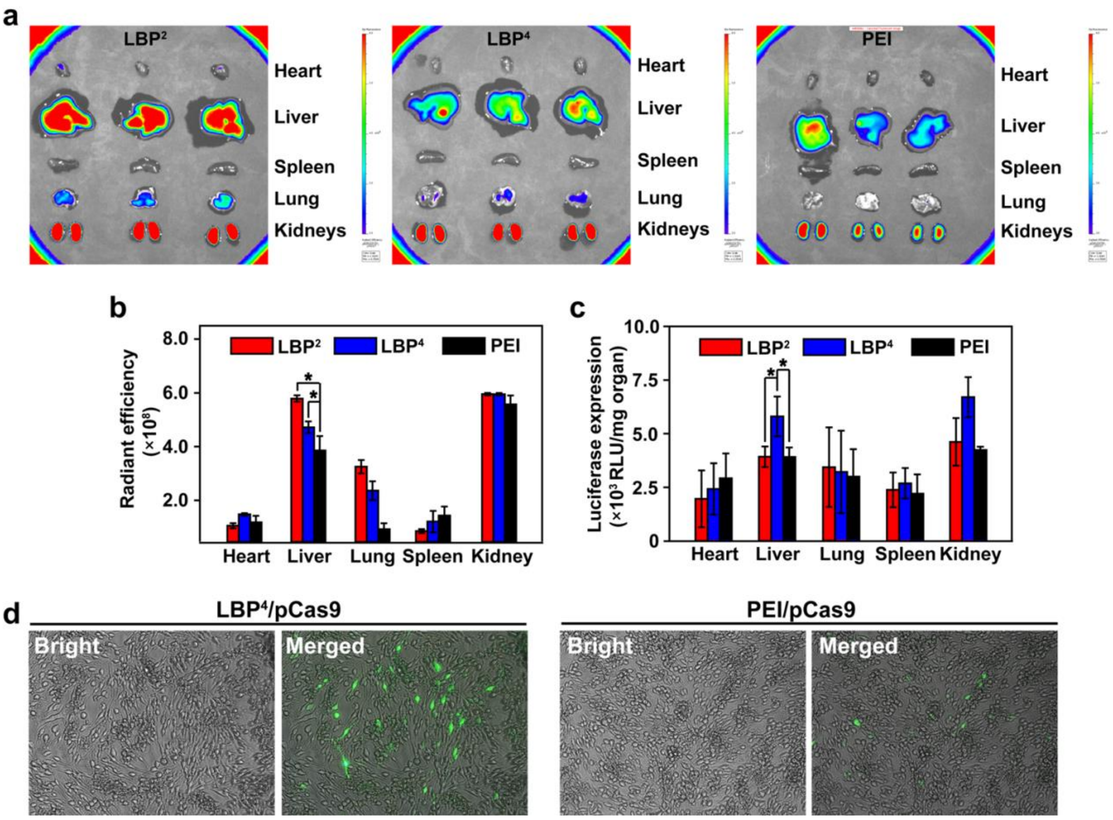

Figure S7. a) Bio-distribution of the LBP^2/pDNA, LBP^4/pDNA and PEI/pDNA complexes in organs (heart, liver, spleen, lung and kidneys) of mice (n = 3) at the 4th hour after tail vein injection. b) The statistical assay of fluorescent signals of the LBP^2/pDNA, LBP^4/pDNA and PEI/pDNA complexes in organs (heart, liver, spleen, lung and kidney) of mice at 4 h after tail vein injection (mean ± SD, n = 3, *$p < 0.05$). c) Luciferase expression of the LBP^2/pDNA, LBP^4/pDNA and PEI/pDNA complexes in organs (heart, liver, spleen, lung and kidney) at the 12th day after tail vein injection (mean ± SD, n = 3, *$p < 0.05$). d) Bright field and merged images of GFP expression mediated by the LBP^4/pCas9 and PEI/pCas9 complexes in BEL7402 cells at the 24th hour after transfection.

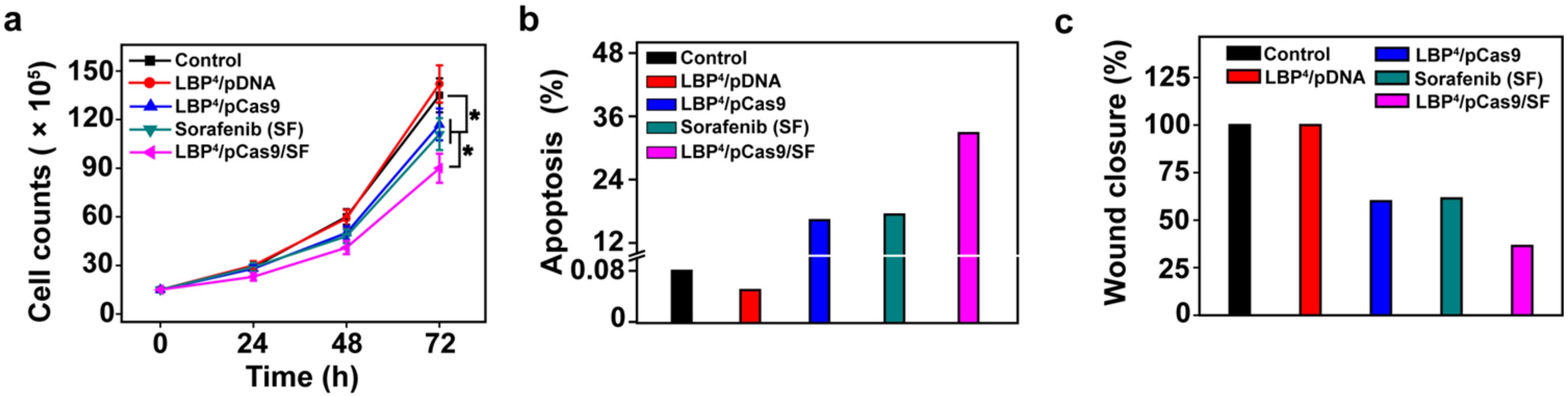

Figure S8. Statistical analysis of different treatment groups in a) proliferation (mean ± SD, n = 3, $*p < 0.05$), and diagrams of b) apoptosis, and c) cellular migration assays.

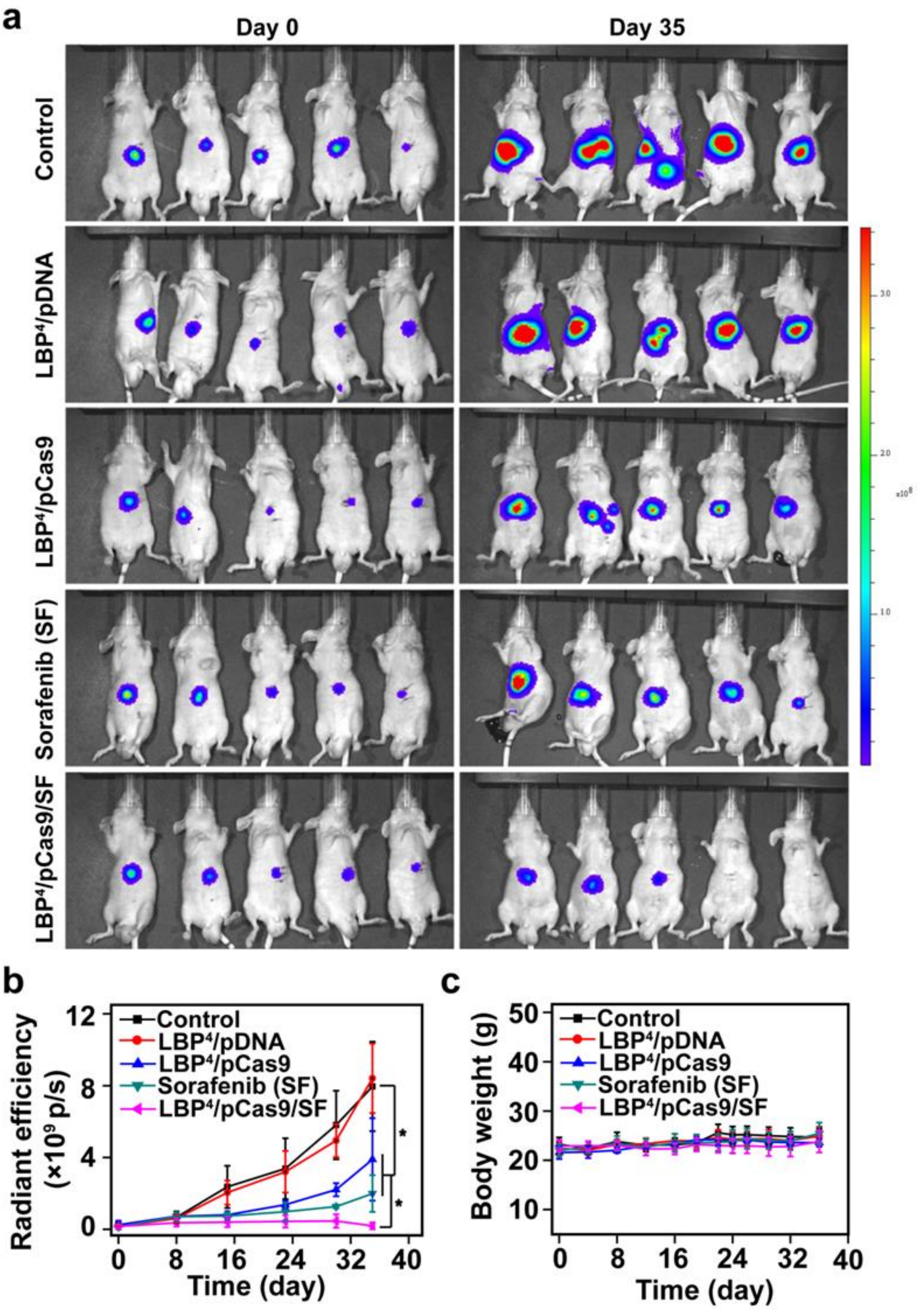

Figure S9. a) Bio-luminescent images of each treatment group at 0th and 35th day (n = 5). b) The radiant efficiency of bio-luminescence from orthotopic tumors in each treatment group from 0 to 35 day (mean ± SD, n = 5, *p < 0.05). c) Average body weights of mice from 0 to 35 day (mean ± SD, n = 5).

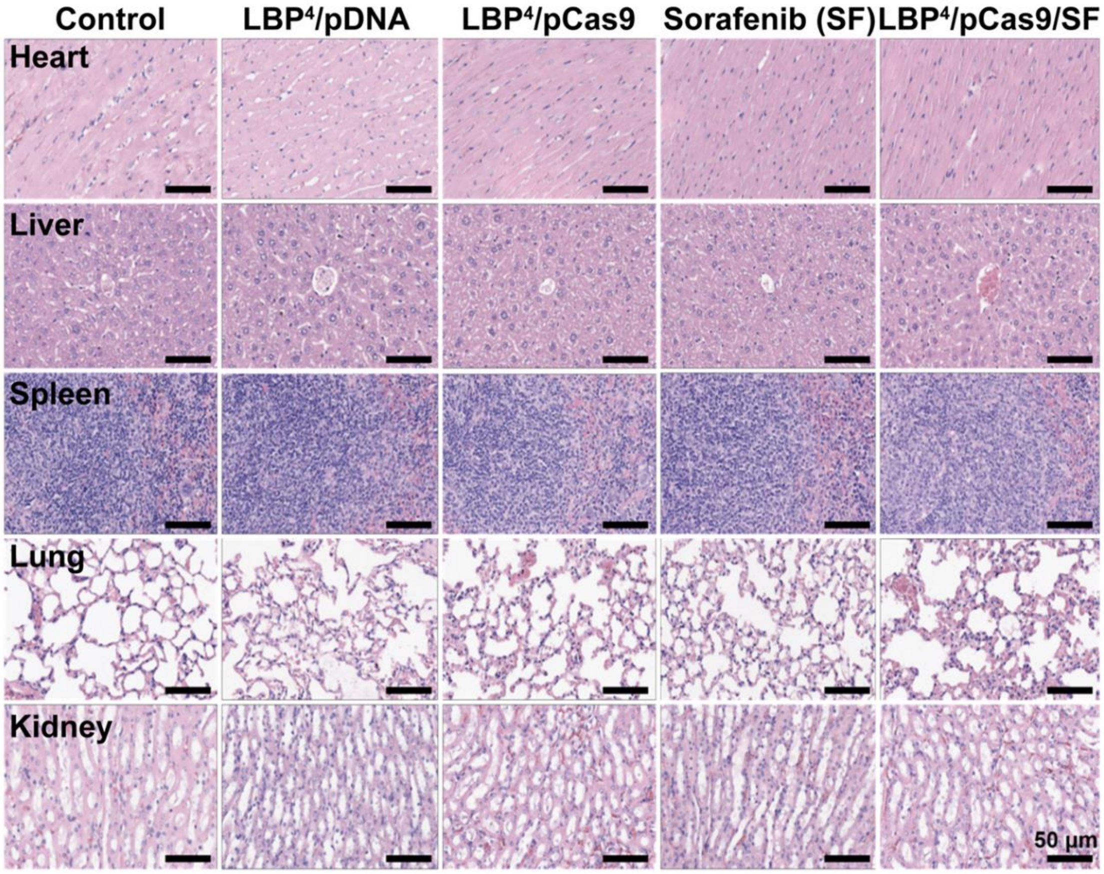

Figure S10. H&E staining of heart, liver, spleen, lung, and kidney from various treatment groups.

Table S1. M_n and PDI of LBPs

Polymer	Molecular Weight (M_n, g.mol^{-1})	Polymer Dispersity Index (PDI)
LBP^2	17526	1.85
LBP^4	20650	1.83

Table S2. Analysis of potential off-target (POT) sites

ID	Sequence (5' - 3')	Position	Number of Off-Target Sites
On target	TCTTGAATGTAGAGATGCGG	chr17	0
POT-1	TCTTGAAaGTAGAaATtCGG	chr2	3
POT-2	TCTTGAAgGgAGAGAgGCGG	chr1	3
POT-3	TCTTGAATtTAGAaATGCGG	chr16	2

Table S3. PCR primers of potential off-target (POT) sites

Potential Off-Target Site	Primers (5' - 3')
POT-1	Forward: GCCTGATGGCCTCCATGTTAT<br>Reverse: CCTCACCCTGTTCCATTGTC
POT-2	Forward: CTCTCCTCGCCTAATGCAGAC<br>Reverse: TGCAGCCTGAGAGTCACAATC
POT-3	Forward: AAGTGAGCTTCTGGCCTTGAT<br>Reverse: AGAGTCTGGGTGCTAGAGCTG
